# Molecular occurrence and genetic diversity of *Ehrlichia canis* in naturally infected dogs from Thailand

**DOI:** 10.1038/s41598-023-47784-4

**Published:** 2023-11-21

**Authors:** Napassorn Poolsawat, Siriphan Sangchuai, Tassanee Jaroensak, Amaya Watthanadirek-Wijidwong, Nitipon Srionrod, Sutthida Minsakorn, Keiichiro Tazawa, Panat Anuracpreeda

**Affiliations:** 1 Parasitology Research Laboratory (PRL), Institute of Molecular Biosciences, Mahidol University, Nakhon Pathom, 73170 Thailand; 2 Department of Parasitology, Faculty of Medicine Siriraj Hospital, Mahidol University, Bangkok, 10700 Thailand; 3 Worldwide Veterinary Service Thailand, Hang Dong, Chiang Mai, 50230 Thailand

**Keywords:** Phylogenetics, Pathogens

## Abstract

Canine monocytic ehrlichiosis is cause by *Ehrlichia canis* resulting in hematologic disorders and severe clinical signs. The aim of this study was to scrutinize the molecular detection and genetic diversity of *E. canis* based on the *trp36* gene in dogs from Thailand’s northern and central regions. A total of 120 dogs blood samples were amplified for *trp36* gene of *E. canis* using the polymerase chain reaction (PCR). Forty-seven out of 120 dog blood samples (39.16%, 47/120) were positive for *E. canis* the *trp36* DNA with 790 bp of PCR amplicon size. The factor significantly associated with *E. canis* infection is animal housing status (*p* < 0.05). Sequence and phylogenetic analysis showed that *E. canis trp36* gene of Thailand isolates was clustered into 1st clade with similarity ranging from 95.65 to 100% together with the US genogroup. The 14 haplotypes of the *trp36* gene shown in TCS network exhibited that haplotype #1–4 was found in Thailand. The entropy analysis of the *trp36* gene illustrated 751 polymorphic sites and 271 entropy peaks of nucleic and amino acid sequences, respectively. Hence, these findings are crucial for better understanding the epidemiology of *Ehrlichia* infection and could be helpful for implementing control measures in Thailand.

## Introduction

*Ehrlichia canis*, an obligately intracellular bacterium transmitted by ticks that affects dogs, causes canine monocytic ehrlichiosis (CME). CME is prevalent in the tropics, particularly in Thailand and Southeast Asia^1–4^. *E. canis* can affect the dog’s monocytes and macrophages, resulting in hematologic disorders and clinical signs like fever, depressive symptoms, anorexia, weight loss, hemorrhage, epistaxis, anemia, thrombocytopenia and even death^5^. The occurrence of CME in dogs has been described in some Thai provinces, including Chiang Mai, Mahasarakham, Buriram, Kalasin, Nakhon Pathom, Songkha and Khonkaen, and can reach up to 36.73 percent, according to results of microscopic examination and PCR assay^6–12^.

The microscopic examination of *E. canis* in Giemsa-stained blood used to diagnose CME has a low sensitivity when parasitemia is low^9, 13^. Serological tests are an alternative method of detection that veterinarians more often use in conjunction with the rapid tests which are commercially available. However, it takes a few weeks for antibodies to occur. When diagnosing infections, particularly in laboratories, the molecular method by polymerase chain reaction (PCR) is reliable and frequently used. It provides high sensitivity and specificity in cases of low parasitemia or early stages of infection in domesticated animals^13, 14^. In *E. canis*, the tandem repeat protein 36 (TRP36) is the immunodominant protein which has been involved with host–pathogen interactions, e.g., adhesion, internalization, actin nucleation and immune evasion^15–17^. TRP36 protein is encoded by the *trp36* gene containing a 5′ end pre-repeat, a tandem repeat and a 3′ end post-tandem repeat regions^15, 18^. Based on TR sequences, *trp36* gene of *E. canis* strains can be divided into four genogroups including United States (US), Taiwan (TWN), Brazil (BR) and Costa Rica (CR)^19–21^. Additionally, novel TR sequences of *E. canis* infection were identified in humans from Costa Rica^21, 22^. Notably, the *trp36* gene exhibited significant variability, rendering it a promising candidate for gene utilization in genetic diversity assessment and clustering^6^. Little is known about *E. canis’s* genetic diversity in Thailand^1, 2, 7, 8^. Therefore, the aim of this study was to scrutinize the molecular detection and genetic diversity of *E. canis* based on the *trp36* gene in dogs from Thailand’s northern and central regions. A bioinformatics sequence analysis was also used to provide more information on the genetic profile of *E. canis* populations in Thailand in comparison to those found in other nations around the world.

## Results

### Occurrence of *E. canis* infection and risk factor analysis

Forty-seven out of 120 samples (39.16%) were positive for *E. canis trp36* gene detected by PCR. The size of PCR product of *E. canis trp36* Thailand sequence was 790 bp. Seven DNA sequences were deposited in GenBank, and accession numbers are provided in Table 1. The results of the univariate analyses regarding the overall *E. canis* infection detected by PCR in association with sex, age, tick infestation and animal housing status are shown in Table 2. The results showed that only animal housing status factor showed higher risk of *E. canis* infection in free roaming group than the dog living in owner house with statistically significant association (*X*^2^ = 11.831, *p* = 0.00058), while the remaining three factors exhibited no statistically significant association as shown in Table 2.

**Table 1:** The *E. canis* nucleotide sequences amplified in Thailand isolate were deposited in the GenBank database.

Regions	Provinces	Districts	Animal ID	GenBank accession numbers
North	Mae Hong Son	Pai	MHS1	OP748419
			MHS2	OP748420
			MHS3	OP748421
Central	Nakhon Nayok	Ban Na	NN1	OP748407
		Muang Nakhon Nayok	NN2	OP748418
		Pak Phli	NN3	OP748408
		Pak Phli	NN4	OP748409

**Table 2:** Factors associated with *E. canis* infection detected by PCR assay.

Parameters	% Occurrence (No. of positive/No. of samples)	Odds ratio (95% CI)	*X*2 (df)	*p-*value
Sex				
Male	22/48 (45.83%)	1.59 (0.75–3.36)	1.4923 (1)	0.2219
Female	25/72 (34.72%)	0.63 (0.3–1.33)		
Total	47/120 (39.16%)			
Age				
Juvenile (< 1 year old)	8/24 (33.33%)	0.73 (0.29–1.87)	0.4293 (1)	0.8068
Adult (1–5 years old)	37/91 (40.67%)	1.3 (0.54–3.12)		
Old (> 5 years old)	2/5 (40.0%)	1.04 (0.17–6.45)		
Total	47/120 (39.16%)			
Tick infestation				
None observed	21/62 (33.87%)	0.63 (0.3–1.32)	1.5098 (1)	0.2192
Tick infestation	26/58 (44.82%)	1.59 (0.76–3.32)		
Total	47/120 (39.16%)			
Animal housing status				
Owner dog	12/54 (22.22%)	0.25 (0.11–0.57)	11.831	0.00058 *
Roaming dog	35/66 (53.03%)	3.95 (1.77–8.82)		
Total	47/120 (39.16%)			

*X^2^, Chi-square test, df, degree of freedom, CI, Confidence interval, PCR, polymerase chain reaction.

### Sequence analysis of *E. canis trp36* gene

**Figure 1:**
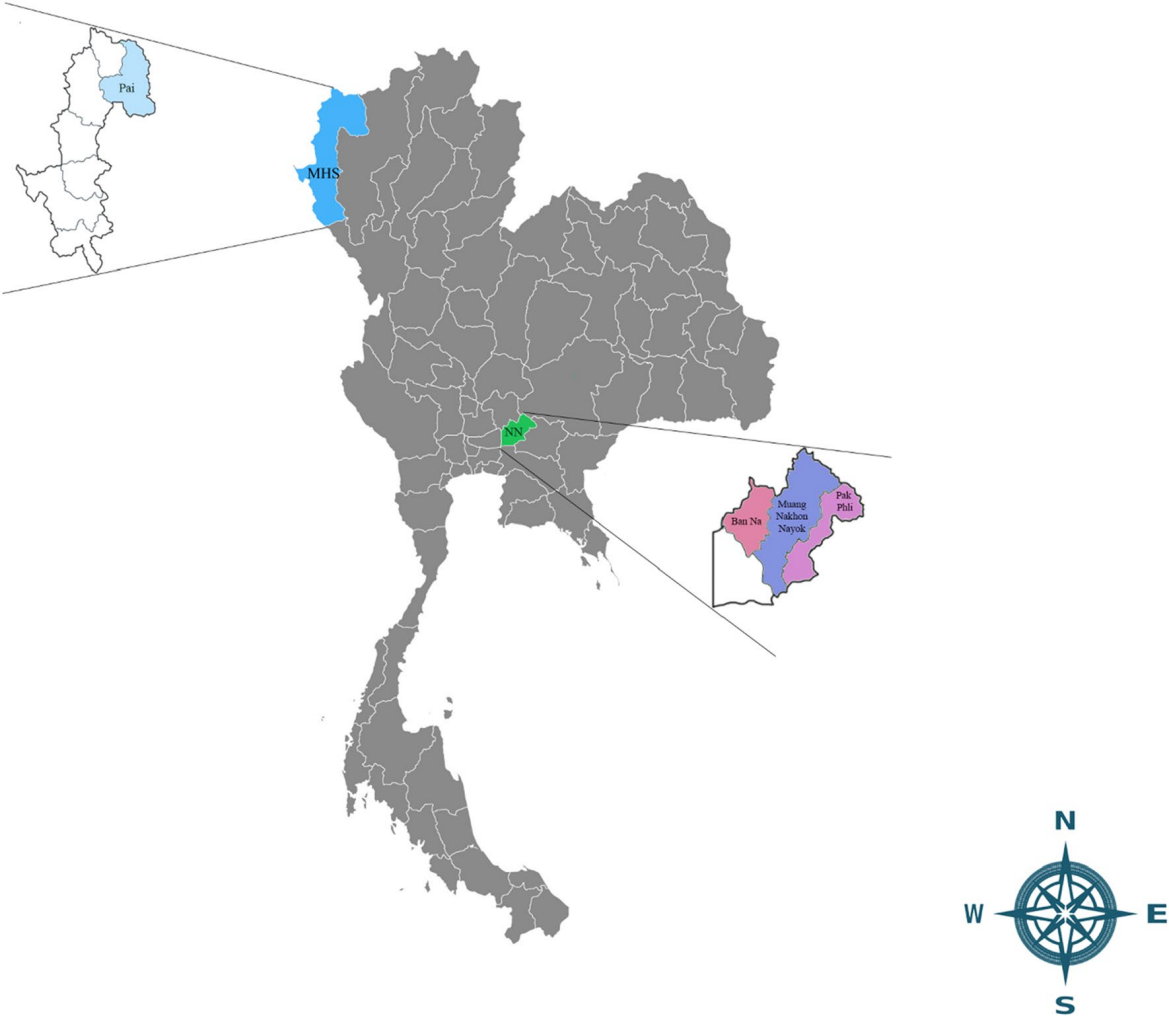
Geographical location of Mae Hong Son and Nakhon Nayok provinces where canine blood samples were collected. Legends indicate the detection of *E. canis trp36* gene Thailand sequences identified in dogs from Pai district in Mae Hong Son (MHS) province and Ban Na, Muang Nakhon Nayok and Pak Phli districts in Nakhon Nayok (NN) province.

*E. canis trp36* sequences was divided into three regions: pre-tandem (427 bp), tandem (27 bp repeat units) and post-tandem repeat regions (none of *trp36* Thailand sequence contained this region due to short sequence amplification). All sequences can be divided into four genogroups including the United States (US), Costa Rica (CR), Brazil (BR) and Taiwan (TWN) (Fig. 1).

### Phylogenetic and similarity analysis of *E. canis* and *trp36* gene sequences

**Figure 2:**
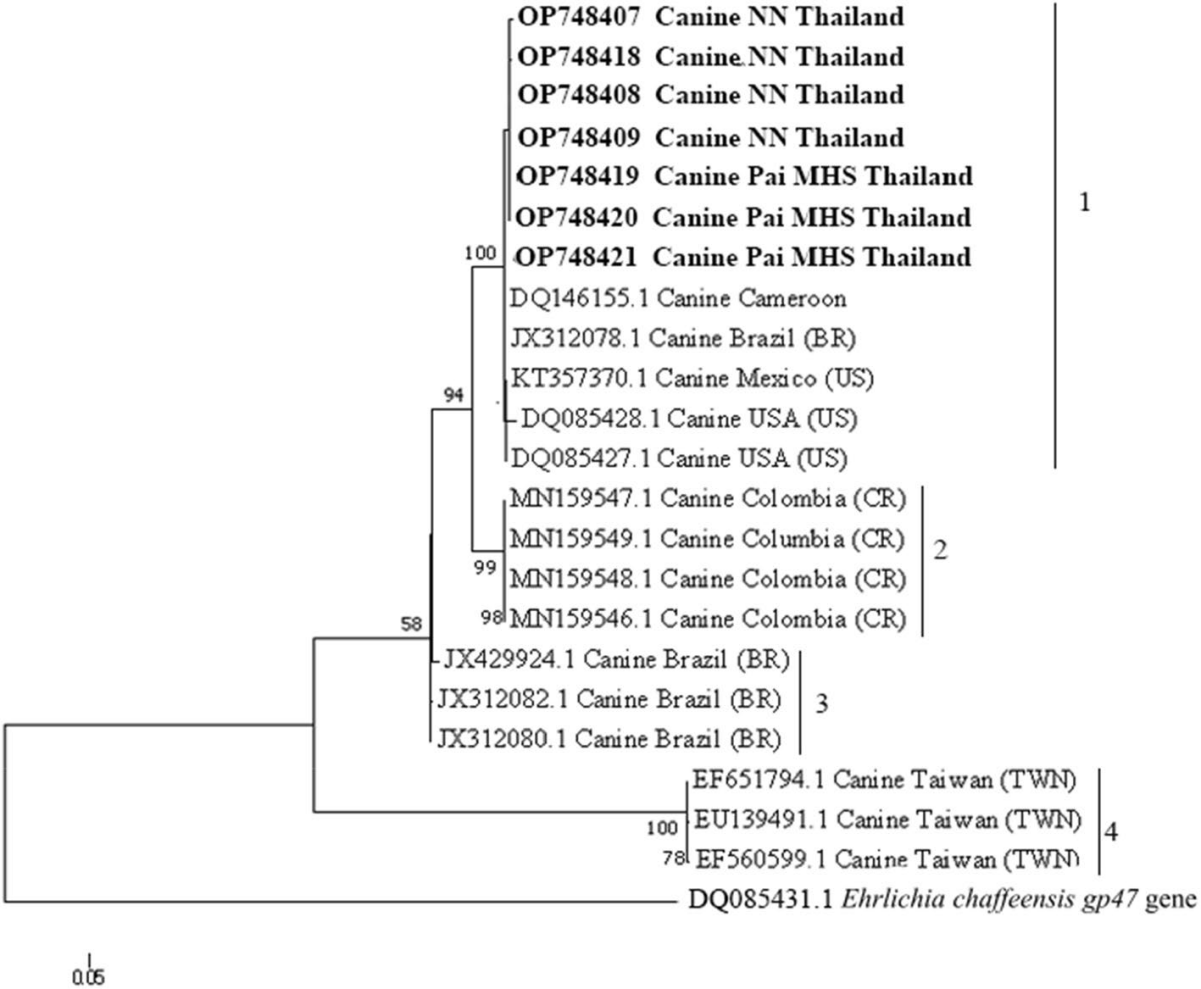
A maximum likelihood phylogenetic tree relationship of *E. canis trp36* gene sequences in this study (boldface) and those obtained from GenBank database. The numbers on each node correspond to the bootstrap analysis of 1000 replicates. The GenBank accession numbers of the sequences used in the phylogenetic trees are also demonstrated. A sequence of *Ehrlichia chaffeensis gp47* gene is used as an outgroup. The scale measures the number of substitution per site.

Seven sequences of *E. canis trp36* gene obtained in this study were aligned with 19 other sequences taken from the GenBank including sequences from USA, Cameroon, Brazil, Mexico, Taiwan and Colombia. The phylogenetic tree of the *trp36* gene was classified as 4 clades (designated as clade 1–4). Our Thailand sequences detected in this work were positioned in 1st clade close to the US genogroup, while clades 2, 3 and 4 were consisted of the sequence from Columbia, Brazil and Taiwan (Fig. 2). The total similarity of Thailand sequences was 95.65–100% (1st clade), while that of the sequences within each clade was 88.58–100% (1st clade, US genogroup) and 97.81–100% (2nd clade, Costa Rica genogroup), 91.72–100% (3rd clade, Brazil genogroup) and 87.25–100% (4th clade, Taiwan genogroup) as shown in Table 3. Additionally, the nucleic acid substitution rate in *trp36* gene sequences among *E. canis* was analyzed by the Tamura and Nei mode as shown in Table 4.

**Table 3:** Similarity of the *E. canis trp36* gene sequences as examined in canine samples in Thailand and other countries.

	Clade	1												2				3			4		
	Acc. No	1	2	3	4	5	6	7	8	9	10	11	12	13	14	15	16	17	18	19	20	21	22
1	OP748407	100.0																					
2	OP748418	97.61	100.0																				
3	OP748408	97.62	98.54	100.0																			
4	OP748409	97.99	98.91	99.65	100.0																		
5	OP748419	97.99	98.91	99.65	100.0	100.0																	
6	OP748420	97.99	98.91	99.65	100.0	100.0	100.0																
7	OP748421	95.65	96.62	96.81	97.19	97.19	97.19	100.0															
8	DQ146155	93.99	95.35	96.22	96.22	96.22	96.22	98.89	100.0														
9	JX312078	93.41	94.81	95.73	95.73	95.73	95.73	98.47	91.98	100.0													
10	KT357370	93.00	94.38	95.28	95.29	95.29	95.29	97.95	91.63	91.33	100.0												
11	DQ085428	93.20	94.57	95.28	95.28	95.28	95.28	98.14	89.21	88.58	89.39	100.0											
12	DQ085427	93.20	94.57	95.47	95.47	95.47	95.47	98.14	92.62	92.08	96.84	89.91	100.0										
13	MN159547	81.69	81.92	80.88	80.88	80.88	80.88	85.21	60.46	60.14	60.01	71.87	60.84	100.0									
14	MN159549	81.46	81.69	80.66	80.66	80.66	80.66	84.99	54.86	59.78	59.81	71.66	60.65	98.02	100.0								
15	MN159548	81.46	81.69	80.66	80.66	80.66	80.66	84.99	65.92	64.04	65.14	71.66	65.35	97.94	97.81	100.0							
16	MN159546	81.46	81.69	80.66	80.66	80.66	80.66	84.99	65.92	64.04	65.14	71.66	65.35	97.94	98.07	99.74	100.0						
17	JX429924	77.67	77.91	77.88	77.88	77.88	77.88	79.64	71.13	69.35	69.99	70.42	70.22	72.02	71.80	71.80	71.80	100.0					
18	JX312082	77.67	77.91	77.88	77.88	77.88	77.88	79.64	65.65	63.95	64.01	68.50	64.22	64.77	64.57	64.18	64.18	91.72	100.0				
19	JX312080	77.67	77.91	77.88	77.88	77.88	77.88	79.64	60.63	59.83	58.20	68.50	59.05	64.28	64.09	64.87	64.87	91.72	94.67	100.0			
20	EF651794	55.74	56.42	56.73	56.10	56.10	56.10	58.60	39.18	35.00	37.48	48.42	38.62	43.26	43.00	46.40	45.88	55.87	53.51	49.83	100.0		
21	EU139491	55.74	56.42	56.73	56.10	56.10	56.10	58.60	27.86	29.42	31.78	48.42	32.73	37.19	33.96	43.74	43.20	55.87	52.26	46.78	91.27	100.0	
22	EF560599	55.74	56.42	56.73	56.10	56.10	56.10	58.60	36.64	34.27	37.14	48.42	39.89	38.25	37.45	44.82	44.29	55.87	52.06	48.11	90.86	87.25	100.0
%Similarity

**Table 4:** The nucleic acid substitution rate in *E. canis trp36* gene sequence.

	A	T/U	C	G
A	–	10.7786	6.5662	**8.5064**
T/U	8.6038	–	**7.1203**	3.6010
C	10.1820	**13.8321**	–	4.9541
G	**13.5659**	7.1945	5.0950	–

Each entry is the probability of substitution (r) from one base (row) to another base (column).

Rates of different transitional substitutions are shown in bold and those of transversional substitutions are shown in italics. The maximum Log likelihood for this computation was -4052.322.

### Haplotype diversity analysis

**Figure 3:**
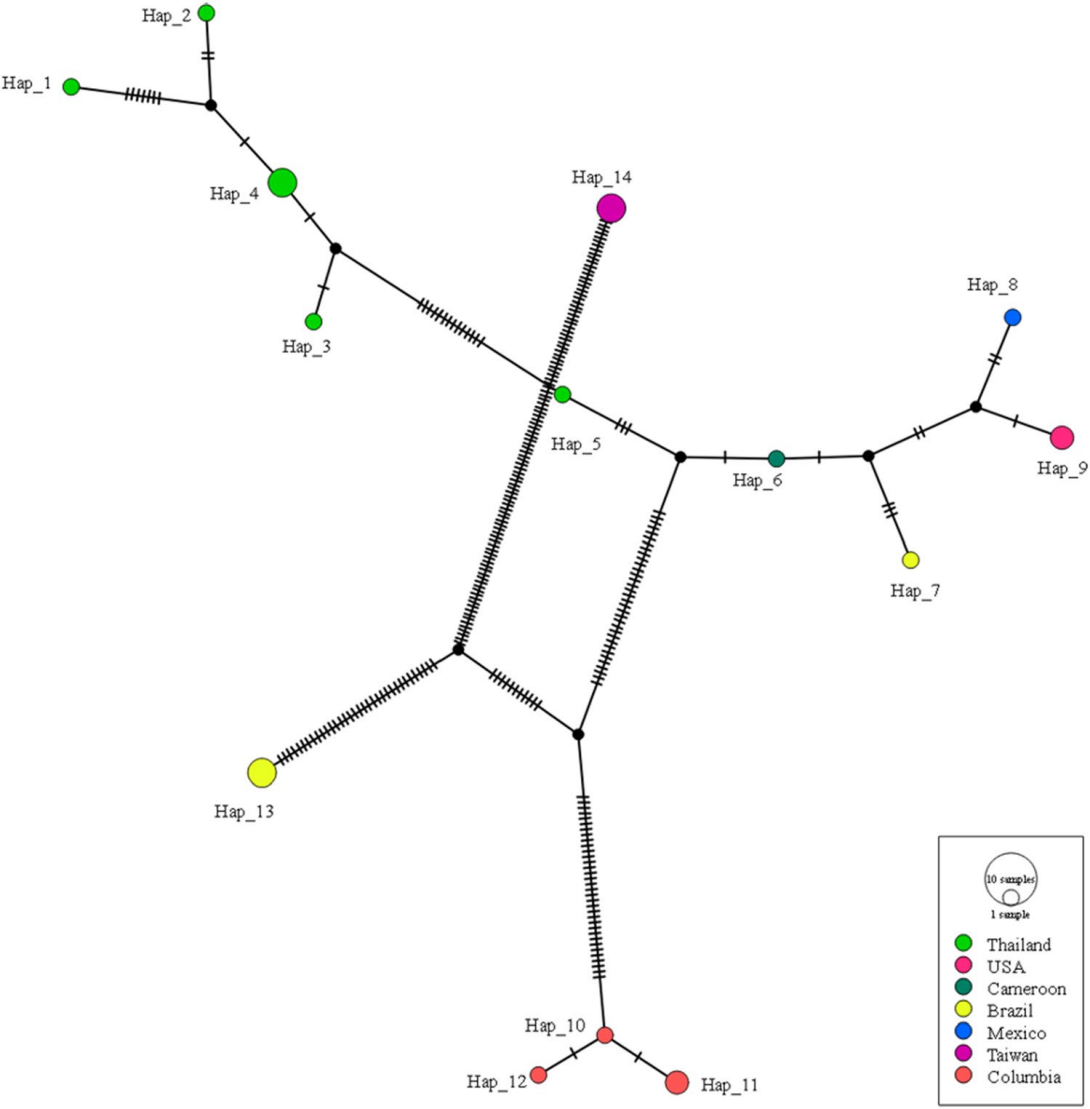
A haplotypeTCS network based on the *E. canis trp36* gene sequence isolated from Thailand and worldwide. Small traits between a haplotype and another represent mutational occurrence. The black circles are the intermediated traits caused by the single nucleotide polymorphism (SNP).

The haplotype networks of *E. canis trp36* gene sequences were constructed from a TCS network tool (Fig. 3). The 14 haplotypes of this gene shown in TCS network exhibited that haplotype #1–4 was found in Thailand, and the rest of the haplotypes were detected in other countries including USA, Cameroon, Brazil, Mexico, Taiwan and Colombia (Fig. 3 and Table 5).

**Table 5:** Polymorphism and genetic diversity of *E. canis trp36* gene sequences as examined in canine samples in Thailand and other countries.

Gene	size (bp)	N	VS	GC%	h	Dh (mean ± SD)	π (mean ± SD)	*K*
*trp36*	790	22	221	34.8	14	0.952 ± 0.026	0.0005594 ± 0.02365	80.18

N, number of analyzed sequence; VS, number of variable sites; GC, G × C content; h, number of haplotypes; Dh, diversity of haplotypes; SD, standard deviation; π, nucleotide diversity (per site); *K*, average number of nucleotide differences.

### Entropy analysis

**Figure 4:**
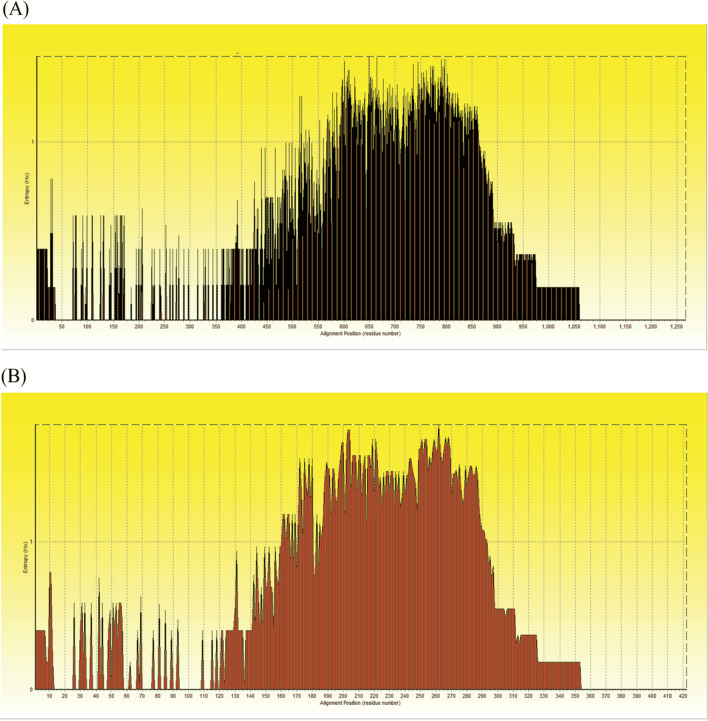
Entropy H_(x)_ analysis of *E. canis trp36* sequence. Entropy plot of multiple nucleic acid sequence alignment of *trp36* genes (**A**). Entropy plot of multiple amino acid sequence alignment of *trp36* gene (**B**). The red peaks refer to high variation at each position of the nucleic (**A**) and amino (**B**) acid sequences.

The entropy analysis of nucleotides revealed that the post-tandem region of *trp36* sequences showed 751 polymorphic sites with entropy values ranged between 0.18491 and 1.46376 (Fig. 4A). Entropy analysis of amino acid sequences was conducted using the TRP36 amino acid sequence alignments. The charts exhibited 271 high entropy peaks for the TRP36 value ranging from 0.18491 to 1.75496 (Fig. 4B).

## Discussion

In Thailand, canine monocytic ehrlichiosis (CME) caused by *E. canis* is a serious tick-borne disease causing severe clinical infection in dogs resulting in death^1–10, 12–14^. Some dogs show healthy appearance, but *E. canis* infection can be detected by PCR screening due to early phase of infection and low parasitemia level^9, 13^. TRP36 protein of *E. canis* encoded by *trp36* gene can elicit in the earliest acute-phase antibody response and involves in host–pathogen interaction^23^. This study is the first report that revealed the infection rate, molecular characteristics and genetic diversity of *E. canis* in dog blood samples in Mae Hong Son and Nakhon Nayok provinces in Thailand. The molecular detection exhibited that of the dogs sampled, 39.16% (47/120) was positive for *E. canis trp36* gene. The occurrence of *E. canis* in this study also agrees with previous reports in Thailand; for instance, 33% in Bangkok^24^, 36% in Chiang Mai, Nonthaburi and Chonburi provinces^2^ and 36.1% in Chiang Mai provinces^6^ By contrast, in Colombia, *E. canis* was found in 11.67% of sampled dogs^25^. The results of univariate analyses indicated that sex and age were not significant to the *E. canis* infection and our results were in line with previous reports of Tazawa et al.^13^ and Mitpasa et al.^12^. For tick infestation factor, the non-significant *p*-value (*p* = 0.219) indicates that there is no statistically significant difference in the frequency of *E. canis* between dogs parasitized by ticks and those without ticks. Most of dogs in this study appear subclinical infection that were recruited for neutralization from different areas. In previous study, Paulino et al.^26^ who revealed that climate change of study area can affect biological growth of *Rhipicephalus sanguineus* which are the vector of *E. canis*^26^. *R. sanguineus* has a life cycle with three-host stages and seeks a new host for a blood meal after each of its three molts, but the pathogens have already transmitted to the infected host. Additionally, dogs living in the shelter or free roaming have higher risk for *E. canis* infection than dogs living with owner significantly (*p* = 0.00058) which is consistent with other studies reported by Mitpasa et al.^12^ and Navarrete et al.^27^.

Although the genetic diversity of *E. canis* strains based on the *trp36* gene has been characterized to 4 genogroups in several countries^19, 27^. There is very little information regarding the genetic diversity and phylogenetic analysis of *E. canis trp36* gene in Thailand so far. The phylogeny analysis of *E. canis trp36* gene Thailand isolates showed totally only one clade with other strains. Bootstrap values in the phylogenetic tree in this study were 78-100% of bootstrap values, which are in line with a majority-rule consensus tree of 1000 replicates for each alignment^28, 29^. The results showed that the genetic diversity and phylogenetic proximity of the *E. canis trp36* gene to the US sequences (US genogroup) were evident from the conserved nucleotide sequence TACTGAAGATTCTGTTTCTGCTCCAGC, which translated to the amino acid sequence TEDSVSAP in the tandem repeat region. This classification grouped Thai samples together with other sequences from the US genogroup in the same clade, showing a similarity range of 88.58–100%. Comparatively, the US genogroup displayed less diversity within the group when compared to the other genogroups in the TCS network. The main differing conserved region were classified by the tandem repeat region of the *E. canis trp36* gene. This finding was similar to previous study in Nonthaburi, Chonburi and Chiang Mai provinces of Thailand reported by Poolsawat et al.^2^ and Nambooppha et al.^6^. This finding indicated the phylogenetic proximity of *E. canis trp36* gene circulating in both different countries and Thailand. Our finding is similar to the previous studies reported by da Costa et al.^30^ and Kaewmonkol et al.^24^.

The *trp36* gene distinguishes itself as an appropriate genotyping marker for *E. canis* strains due to its alleles encoding distinct TR amino acid sequences of TRP36. Its utility extends to the assessment of genetic diversity among E. canis isolates, revealing pronounced variations in TR sequences and/or TR numbers across diverse geographic regions^19, 20, 31^. The most preserved TR in *E. canis* strains worldwide is TEDSVSAPA from the US genotype, and a similar preservation is observed in Taiwan genogroups with different N-terminal pre-TR regions^17, 19^. A novel Brazilian genotype has been reported with a different tandem repeat sequence (ASVVPEAE) in dog samples in Brazil. However, some dog samples in Brazil exhibit a pre-TR region similar to US genogroups^17, 20^. A novel genotype consisting of one TR with the sequence EASVVPAAEAPQPAQQTEDEFFSDGIEA was reported in the Costa Rica (Cr) genogroup^21^. Moreover, TR sequences of EASVVPAAEAPQPAQQTEDEFFSDGIEA and EASVVPAAEAPQPAQQTEDEFFSDGIE amino acid sequences were identified in humans from Costa Rica^22^. In many studies, some isolates in the same country were classified into different genogroups depending on their sequences. For instance, in the study of Turkish isolates of *E. canis*, it was reported that the Turkish isolate sequences were segregated into four distinct genogroups: US genogroups I and II, Brazilian genogroup, and Costa Rica-Turkey genogroup. Seven *E. canis* Turkish isolates and *E. canis*-human Costa Rica were placed in a new genogroup designated in this study as Costa Rica-Turkey genogroup^22^.

In this study, our Thailand samples were genetically conserved and closed to the US genogroup sequences as shown in TCS network and shared genetic traits with other sequences as retrieved previously worldwide. The Taiwan and Brazil genogroups contain single-nucleotide polymorphism (SNP) trait different from Thailand sequences related to the different of nucleotide base and translated amino acid in tandem repeat and post-tandem repeat regions of the *trp36* gene. The high SNP variations, which are linked to a high number of nucleotide and amino acid variables, are shown by the high entropy values and polymorphic sites. The lower entropy values reveal that each sequence contains few SNP variants^32^.

The genetic diversity observed in the *trp36* gene, particularly in the tandem repeat region, has revealed a potential novel target for organism genotyping. This study’s findings contribute to our understanding of *E. canis*’ genetic diversity and highlight the importance of further research to analyze genetic variations in *E. canis* strains worldwide. TRP36 protein, encoded by the *trp36* gene (DQ146154 in GenBank)^18^, exhibits distinct expression patterns within the dense-cored morphological variant of *Ehrlichia*. In this form, the protein is both exposed on the cell surface and secreted^15^. TRP36 protein of *E. canis* represents an immunodominant protein, playing a significant role in host-pathogen interactions and triggering the earliest acute-phase antibody response during the disease progression^15^. Its recognition as a surface protein early in the infection process makes TRP36 a promising candidate for diagnostic tools and vaccine development^15, 23^.

## Conclusions

This study is the first report regarding a molecular occurrence and genetic diversity of *E. canis* in canine samples from Thailand’s Mae Hong Son and Nakhon Nayok provinces. Our results revealed that the diversity of *E. canis trp36* gene is genetically conserved in Thailand and worldwide. These results may help to clarify the molecular phylogeny and diversity of the *trp36* genes of *E. canis* Thailand strains. Hence, our finding may be useful in immunodiagnostic tools and vaccination for CME.

## Methods

### Sample population

This study was conducted during October 2022 to March 2023. A total of 120 blood samples from canine shelters in the north (17 dogs from Pai district; Mae Hong Son province, 19° 22′ 51.222″ N latitude, 98° 26′ 40.1064″ E longitude) and central (103 dogs from Ban Na, Muang Nakhon Nayok, Pak Phli district; Nakhon Nayok province, 14° 13′ 7.608″ N latitude, 101° 18′ 24.84″ E longtitude) regions of Thailand, were used in this study (Fig. 1). The sample sizes were calculated using the formula based on the equation, *n* = *t*^2^ × *p *(1 *− p*)*/m*^2^, inserting the following values: the prevalence (*p*) of *E. canis* infection among dogs in Thailand, a 95% confidence level (*t*) and 5% margin of error (*m*)^1, 13^.

### Collection of blood samples

Approximately three ml of whole blood samples were obtained from the cephalic or lateral saphenous veins of each animal, collected in EDTA-tubes (BD Vacutainer^®^, USA) and kept at − 20 °C. Additionally, licensed veterinarians carried out the processes of animal restraint and blood sample collection.

### DNA extraction and PCR amplification of the *trp36* gene of *E. canis*

Genomic DNA of *E. canis* was extracted from dogs’ blood samples using a DNA Extraction Kit (OMEGA, bio-tex, USA) according to the protocol of Junsiri et al.^33–35^, Poolsawat et al.^1, 2^ and Watthanadirek et al.^36^ with some modifications. Briefly, the DNA sample was eluted in 30 µl MiliQ water and concentration of purified DNA sample was defined with NanoDrop™ 2000 Spectrophotometers (Thermo Scientific™, USA) at the 260/280 and 260/230 ratios. Finally, the aliquots were stored at − 20 °C until further use. The *trp36* gene was amplified by single PCR using the specific primers: TRP36F 5′-ATGCTACTTTTACTAATGGGTTATTGT-3′ and TRP36R 5′-GTACAACATGTTAAGAATATCAG-3′^24^ according to the protocol of Poolsawat et al.^2^. For PCR reaction, 50 ng of purified DNA template was added in a total volume of 25 μl of reaction mixture containing 0.2 μM of each primer, 200 μM of each deoxynucleoside triphosphate (dNTPs), 1 × phusion HF buffer, nuclease free water and 0.5 U Phusion^®^ High-Fidelity DNA Polymerase (NEW ENGLAND BioLabs^®^Inc, USA). The thermocycling protocol for the *trp36* gene was carried out with the conditions: 98 °C for 3 min followed by 35 cycles at 98 °C for 60 s, 56 °C for 60 s, 72 °C for 90 s, and 72 °C for 5 min. The PCR amplicon was stained with FluoroStain™ DNA Fluorescent Staining Dye (SMOBIO^®^, Taiwan). PCR products were visualized with gel electrophoresis using 1% agarose gel under UV illumination and photographed. A 100 bp DNA Ladder M (SMOBIO^®^, Taiwan) was used as a standard for defining the molecular mass of PCR products.

### Molecular cloning and sequencing of *E. canis trp36* gene

The purified PCR product was cloned into the pGEM^®^-T Easy vector (Promega, USA). The ligation product was transformed into the *Escherichia coli* strain DH5-alpha cells (Invitrogen, USA). Then, the transformed *E. coli* cells were cultured on the Luria Bertani (LB) medium agar plate supplemented with ampicillin (100 μg/ml) and X-GAL (20 mg/ml). After incubation at 37 °C overnight, the white colonies were selected and grown in LB medium containing ampicillin for overnight. Finally, the recombinant plasmid (pGEM^®^-T-*trp36*) was extracted from the competent cell using the Presto™ Mini Plasmid Kit (Geneaid, Taiwan) following the manufacturer’s instructions, and analyzed for accurate sized inserts by agarose gel electrophoresis. The presence of *trp36* insert was confirmed by Sanger sequencing. All sequences were analyzed by BLAST (The National Center for Biotechnology Information, NCBI, http://www.ncbi.nlm.nih.gov/ BLAST), and deposited in the GenBank database.

### Phylogenetic tree analysis

The *E. canis trp36* gene sequences were aligned with Muscle algorithm, and genetic inference was carried out with phylogenetic tree which was reconstructed using the maximum likelihood (ML) as implemented in the MEGA software v.7.0.26^37^. Bootstrap analysis with 1000 repetitions was used to assess the reliability of the branching pattern of the ML trees^38^. The evolutionary distance was analyzed by the Hasegawa–Kishino–Yano model^39^. The similarity of nucleotide sequences was evaluated by a sequence identity matrix in Bioedit software v.7.0.5.3^40^.

### Analysis of haplotype diversity

The sequences alignment of *E. canis trp36* gene was employed to evaluate the nucleotide diversity (π), diversity of haplotypes (Dh), number of haplotypes (h), and the average number of nucleotide differences (*K*), using the DnaSP v.6.12.03^41^. All sequences were subjected to the popART program^42^ to construct the TCS Network^43^.

### Entropy analysis

Entropy estimation was employed to ascertain the variability of the nucleotide and amino acid sequences of *E. canis*. The *E. canis trp36* nucleotide sequences were translated into amino acid sequences, aligned and analyzed by the entropy (H (x)) plot using Bioedit software v.7.0.5.3^40^.

### Statistical analysis

The demographic factors and the overall infection status were analyzed in relation to the infection using Pearson’s Chi-squared test. The relationship between risk factors and occurence was analyzed using the logistic regression test with *p*-value < 0.05 in SPSS software v. 22.0 (IBM Corp., NY, USA) (IBM Corp., 2013)^44^.

### Ethics statement

All experimental procedures on animals were approved by the Animal Care and Use Committee (IMBMU-ACUC), Institute of Molecular Biosciences, Mahidol University, Thailand. All biological samples were collected with authorized consent form from the canine shelter and hospital. In addition, all methods were performed in accordance with the relevant guidelines and regulations.

## Data Availability

The datasets generated and/or analysed during the current study are available in the GenBank database, and the accession number are OP748407, OP748408, OP748409, OP748418, OP748419, OP748420, and OP748421. The datasets generated during and/or analysed during the current study are available from the corresponding author on reasonable request.
